# Systemic application of 3-methyladenine markedly inhibited atherosclerotic lesion in ApoE^−/−^ mice by modulating autophagy, foam cell formation and immune-negative molecules

**DOI:** 10.1038/cddis.2016.376

**Published:** 2016-12-01

**Authors:** Shen Dai, Bo Wang, Wen Li, Liyang Wang, Xingguo Song, Chun Guo, Yulan Li, Fengming Liu, Faliang Zhu, Qun Wang, Xiaoyan Wang, Yongyu Shi, Jianing Wang, Wei Zhao, Lining Zhang

**Affiliations:** 1 Department of Immunology, Shandong University School of Medicine, Jinan 250012, Shandong, China; 2 Department of Neuroscience, Temple University School of Medicine, Philadelphia, PA, USA

## Abstract

A growing body of evidence demonstrates that autophagy, an evolutionarily conserved intracellular degradation process, is involved in the pathogenesis of atherosclerosis and has become a potential therapeutic target. Here we tested the effect of two inhibitors of phosphatidylinositol 3-kinase, 3-methyladenine (3-MA) and 2-(4-morpholinyl)-8-phenyl-chromone (LY294002), commonly used as inhibitors of autophagy, in atherosclerosis in apolipoprotein E^−/−^ mice. Systemic application of 3-MA but not LY294002 markedly reduced the size of atherosclerotic plaque and increased the stability of lesions in high-fat diet-fed mice as compared with controls. Furthermore, 3-MA had multiple atheroprotective effects, including modulating macrophage autophagy and foam cell formation and altering the immune microenvironment. Long-term treatment with 3-MA promoted oxidized low-density lipoprotein (oxLDL)-induced macrophage autophagy and suppressed foam cell formation and cell viability *in vitro*. Furthermore, systemic application of 3-MA promoted lipid droplet breakdown and decreased apoptosis, most likely associated with autophagy. 3-MA treatment strikingly enhanced the expression of immune-negative molecules such as interleukin 10 (IL-10), transforming growth factor *β* and IL-35, as well as forkhead box P3 (Foxp3), the specific transcriptional factor for regulatory T cells, but did not affect the level of proinflammatory cytokines in the arterial wall. We provide strong evidence for the potential therapeutic benefit of 3-MA in inhibiting atherosclerosis development and improving plaque stability.

Atherosclerosis is a chronic inflammatory and metabolic disease in the wall of large- and medium-sized arteries. Modified low-density lipoprotein (LDL), such as oxidized LDL (oxLDL), triggers the disease by deposition at specific sites of the arterial intima, thereby becoming a crucial stimulator of the innate and adaptive immune system.^1^ In this way, the uptake of modified lipoproteins by macrophages accompanied by defective cholesterol efflux results in foam cell formation, which has an important role in the growth of atherosclerotic plaque and vulnerable plaque.^2, 3, 4^

Macroautophagy (hereafter referred to as autophagy) is a highly conserved lysosomal degradation pathway by which intracellular components, including soluble macromolecules (e.g., proteins and lipids) and dysfunctional organelles (e.g., mitochondria and endoplasmic reticulum) are degraded and recycled to maintain cellular homeostasis.^5^ Accumulating evidence suggests that autophagy, especially macrophage autophagy, has an important role in the pathogenesis of atherosclerosis.^6, 7, 8, 9^ In advanced atherosclerosis, generation of several atherosclerotic factors, such as oxLDL,^10^ 7-ketocholesterol^11^ and reactive oxygen species,^12^ may result in dysfunctional autophagy, thereby leading to plaque development and instability. Basal autophagy has an essential role in anti-atherosclerosis.^13, 14, 15^ Basic autophagy deficiency in macrophages by specific autophage protein 5 knockout accelerated atherosclerotic plaques in high-fat diet (HFD)-fed *ldlr*^−/−^ mice via promoting oxidative stress, plaque necrosis^13^ or inflammasome hyperactivation.^14^ As well, autophagy can enhance the breakdown of lipids in lipid droplets (LDs) and cholesterol efflux from macrophage foam cells and further inhibit atherogenesis.^15^

With the effects of autophagy on atherosclerosis development, pharmacological modulation of autophagy becomes a potential approach to slow the progress of plaque and stabilize vulnerable lesions.^16, 17^ The activators of autophagy, such as rapamycin or everolimus, which augment autophagy via inhibition of mammalian target of rapamycin (mTOR), have been used as add-on therapy to prevent or delay the pathogenesis of atherosclerosis.^18, 19, 20^ Furthermore, stent-based delivery of everolimus selectively cleared macrophages in atherosclerotic plaques by activating autophagy and blocking protein synthesis.^21, 22^ However, another activator of autophagy, imiquimod, had adverse effects: imiquimod-induced macrophage autophagy has been associated with inflammation and plaque progression.^23^ To explore potential therapeutic strategies for atherosclerosis via regulating autophagy, further studies are needed to fully evaluate the feasibility of several related compounds.

3-Methyladenine (3-MA) is a widely used inhibitor of autophagy because of its inhibitory effect on class III phosphatidylinositol 3-kinase (PtdIns3K).^24, 25, 26^ However, 3-MA has an autophagic promotion effect under nutrient-rich conditions based on its inhibitory effect on class I PtdIns3K.^25, 26^ *In vivo*, 3-MA has had promising therapeutic benefit in ameliorating experimental autoimmune neuritis in rat^27^ and controlling Enterovirus 71 infection and pathogenesis.^28^ Moreover, 3-MA can suppress tumor metastasis in an autophagy-independent manner.^29^ However, the role of 3-MA in atherosclerosis development remains to be investigated.

To assess whether 3-MA can affect atherosclerosis, we systemically administered 3-MA to apolipoprotein E (ApoE)^−/−^ mice fed a HFD for 8 weeks and analyzed its effect on atherosclerosis. We also tested the effect of 2-(4-morpholinyl)-8-phenyl-chromone (LY294002), another phosphoinositide 3-kinase (PI3K) inhibitor and commonly used as an autophagy inhibitor.^25^ Administration of 3-MA significantly inhibited the formation of atherosclerotic lesions and enhanced the stability of plaque; LY294002 had no atheroprotective effects. Our data provide strong evidence for the potential therapeutic benefit of 3-MA in atherosclerosis. We explored the potential mechanism of the role of 3-MA in atheroprotection and found multiple mechanisms, including modulating macrophage autophagy and foam cell formation and modifying the immune microenvironment.

## Results

### 3-MA markedly inhibited the development of atherosclerotic lesion in ApoE^−/−^ mice fed an HFD

**Figure 1:**
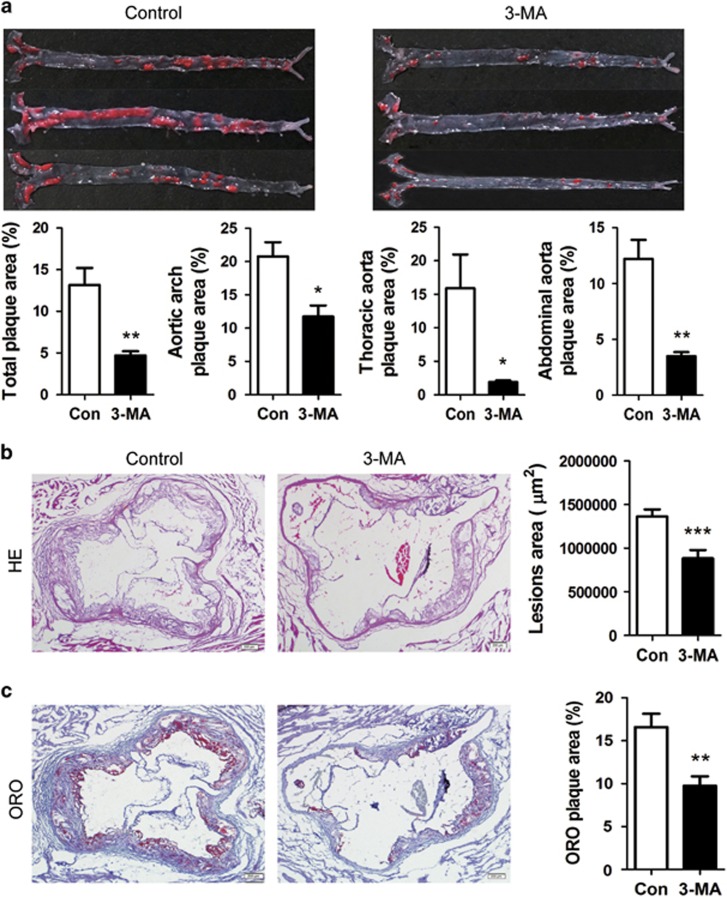
3-MA markedly inhibited the development of atherosclerotic lesion in ApoE^−/−^ mice fed with HFD. (**a**) Representative images of Oil Red O-stained plaque burden (red) in aortas from control mice (6 mice from 14 control mice) and 3-MA-treated mice (6 mice from the 16 3-MA-treated mice). Data are percentage ORO-stained plaque areas in the entire aorta and regions (aortic arch, thoracic aorta and abdominal aorta). (**b** and **c**) Representative hematoxylin and eosin (H&E) staining and Oil Red O staining of cross-sections of aortic root in control mice (*n*=14) and 3-MA-treated mice (*n*=16). Scale bar, 200 *μ*m. Data are mean±S.D. *P<0.05, **P<0.01, ***P<0.001, unpaired two-tailed Student's *t*-test

To investigate potential therapeutic effect of autophagy inhibitor in the development of atherosclerosis, we first tested the effect of 3-MA on atherosclerosis in HFD-fed ApoE^−/−^ mice. Oil Red O staining of aortas showed that mice treated with 3-MA (*n*=16) showed significantly less atherosclerotic lesions in the whole aorta (including aortic arch, thoracic and abdominal regions) compared with phosphate buffer solution (PBS)-treated controls (*n*=14; Figure 1a). Consistently, hematoxylin and eosin (H&E) staining and Oil Red O staining of aortic roots also showed significantly decreased atherosclerotic plaques and lipid content in 3-MA-treated mice (Figures 1b and c). To explore whether other autophagy inhibitors have the same effect, we investigated the impact of LY294002 on the development of atherosclerosis. LY294002 treatment had no significant effect on atherosclerotic lesions (Supplementary Figure S1).

3-MA and LY294002 had no significant side effect on body weight and serum lipid profile including triglycerides (TGs), total cholesterol (TC), LDL and high-density lipoprotein (HDL; Supplementary Table S2). In addition, 3-MA and LY294002 treatment did not damage liver and kidney function (Supplementary Table S3). Of note, 3-MA slightly decreased aspartate aminotransferase (AST) level as compared with controls.

### 3-MA application improved the stability of plaque by decreasing the number of macrophages and apoptotic cells, but increasing the proportion of smooth muscle cell

**Figure 2:**
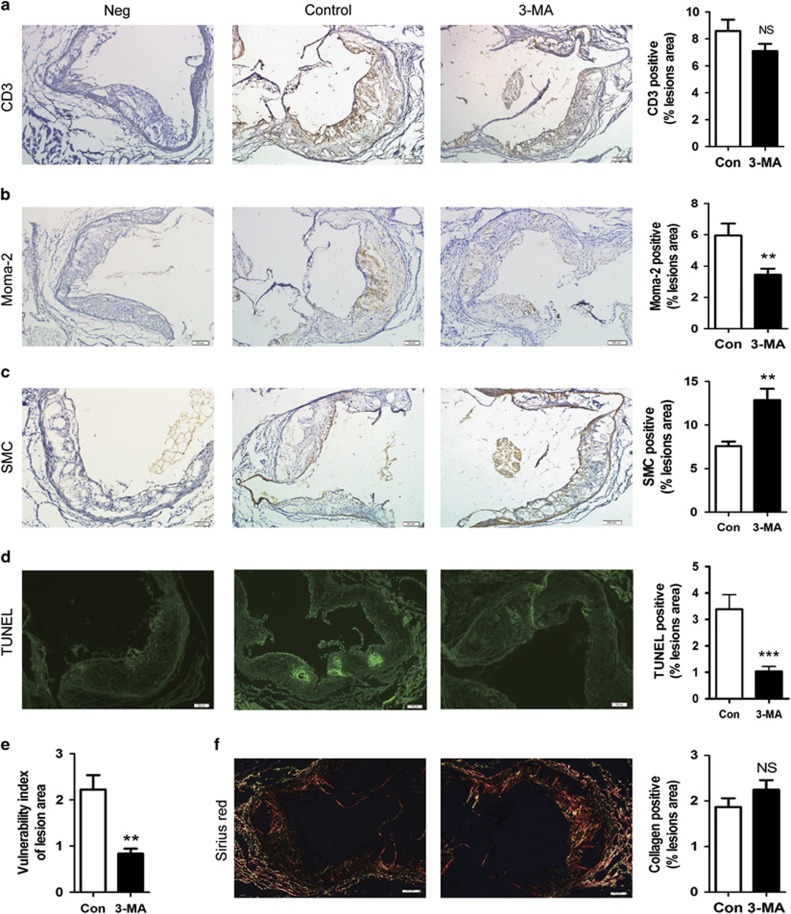
3-MA application improved the stability of plaque. (**a**–**d** and **f**) Cross-sections of aortic root from control mice (*n*=14) and 3-MA-treated mice (*n*=16) stained for T cells (CD3), macrophages (MOMA-2) and smooth muscle cells (αSMC-Actin). Sirius red staining was used to detect collagen fibers. TUNEL staining was used to detect apoptotic cells. Scale bar, 100 *μ*m. (**e**) Analysis of vulnerability index of control mice (*n*=14) and 3-MA-treated mice (*n*=16). Data are mean±S.D. **P<0.01, ***P<0.001, unpaired two-tailed Student's *t*-test

To resolve the question of whether 3-MA affected the stabilization of atherosclerotic plaque, we assessed the proportion of smooth muscle cells (SMCs), infiltrated immune cells (T cells and macrophages) and collagen content in aortic roots by IHC staining and Sirius red staining. 3-MA did not affect CD3 proportion in lesions (Figure 2a), but decreased the number of infiltrated macrophages, presented as elevated MOMA-2 level, as compared with controls (Figure 2b). In parallel, the proportion of SMCs was increased as compared with controls (Figure 2c). Collagen content within the plaque of 3-MA-treated mice was increased but not significantly (Figure 2f). 3-MA treatment decreased the number of TUNEL-positive apoptotic cells (Figure 2d). We calculated the vulnerable index, the ratio of plaque area occupied by lipid components (in macrophages+extracellular lipids) and by fibromuscular components (SMCs+collagen fibers),^30^ and found it significantly lower in mice with 3-MA than controls (Figure 2d), so 3-MA promoted a stabilized phenotype in the lesion area. However, LY294002 application had no significant effect on plaque contents (Supplement Figure S2).

### 3-MA promoted oxLDL-induced macrophage autophagy *in vitro*, but decreased the level of autophagy in plaque *in vivo*

**Figure 3:**
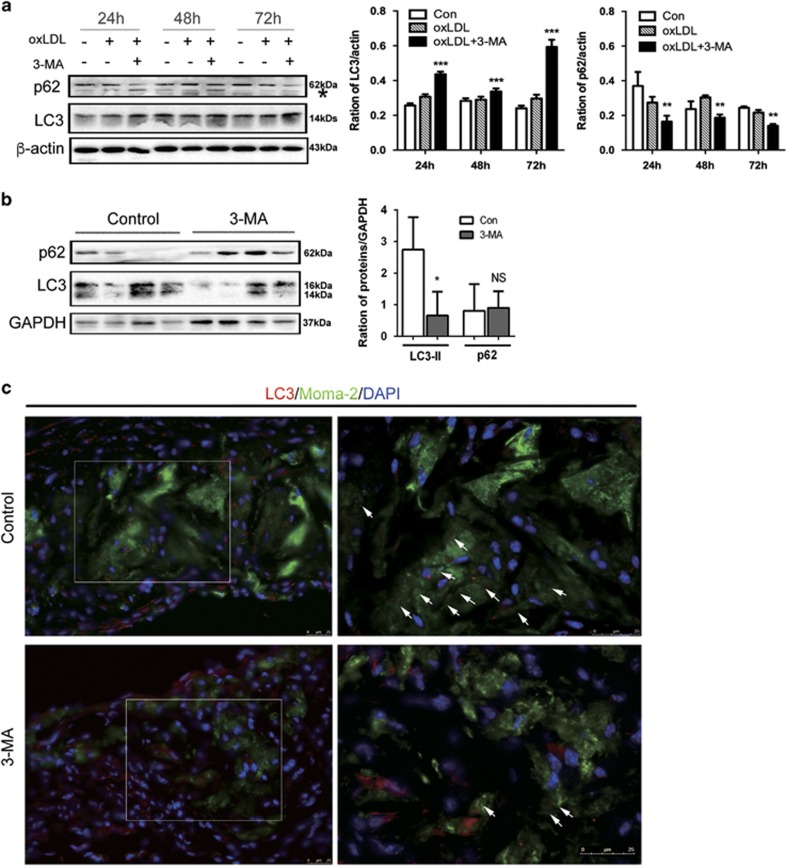
3-MA affected autophagy both *in vitro* and in plaque *in vivo*. (**a**) Cultured peritoneal macrophages were preincubated with 3-MA for 30 min, then stimulated with 25 *μ*g/ml oxLDL. Western blot analysis of autophagy activity at different times after oxidized low-density lipoprotein (oxLDL) stimulation. The asterisk indicates cross-reactive band of p62 antibody. Data are mean±S.D. **P<0.01, ***P<0.001, two-way ANOVA. (**b**) Western blot analysis of LC3 and p62 level in whole-aorta lysates from control mice (4 mice from 14 control mice) and 3-MA-treated mice (4 mice from 16 3-MA–treated mice). Data are mean±S.D. from three independent experiments. *P<0.05, unpaired two-tailed Student's *t*-test. (**c**) Immunofluorescence staining of LC3 co-stained with MOMA-2 in aortic root sections from control mice and 3-MA-treated mice. Right panels of LC3 staining (white arrows; scale bar, 25 *μ*m) are the enlargements of boxed areas in left panels (scale bar, 25 *μ*m)

Although 3-MA is often used as inhibitor of autophagy,^24, 25^ recent research has shown that 3-MA can promote autophagy flux under nutrient-rich conditions with prolonged treatment.^26^ To explore the possible mechanisms by which 3-MA has an anti-atherosclerotic effect, we detected the impact of 3-MA on autophagy *in vitro* and *in vivo*. Because macrophage autophagy has a critical role in atherosclerosis,^8^ we tested the effect of 3-MA on macrophage autophagy during foam cell formation *in vitro*. Cultured primary peritoneal macrophages were stimulated with oxLDL for 24, 48 and 72 h with and without 3-MA. 3-MA significantly increased the protein expression of microtubule-associated protein light chain 3 (LC3), a hallmark of autophagosome formation, and decreased that of sequestosome 1 (SQSTM1, p62), the substrate of autophagosome commonly used to reflect autophagy flux in lesion,^25, 26^ as compared with controls (Figure 3a), which suggests that 3-MA improved autophagy flux in oxLDL-stimulated macrophages. Furthermore, we detected autophagy level in plaque of 3-MA-treated mice and controls. In contrast with *in vitro* results, plaque of 3-MA-treated mice showed decreased LC3-II expression as compared with controls (Figure 3b). We also used immunofluorescence staining to detect the change of LC3-II level in plaque. Despite the existence of nonspecific signals (positive signals with diameter >0.5–1.5 *μ*m, the average diameter of autophagosome in mammals) from other plaque components, such as cellular debris, positive-stained LC3 puncta in macrophage-rich areas of plaque of 3-MA-treated mice were much less than in controls (Figure 3c). Moreover, to determine whether the PI3K signal pathway was affected by 3-MA, we used IHC staining to study the class I PI3K pathway activation by one of its downstream target proteins, the phosphorylated form of ribosomal protein S6 (pS6), in aortic root of 3-MA-treated mice and controls. Level of pS6 was lower with 3-MA than control treatment, although not significantly (*P*=0.0876; Supplementary Figure S3b).

### 3-MA decreased foam cell formation *in vitro* and limited the accumulation of LDs within atherosclerotic plaque *in vivo*

**Figure 4:**
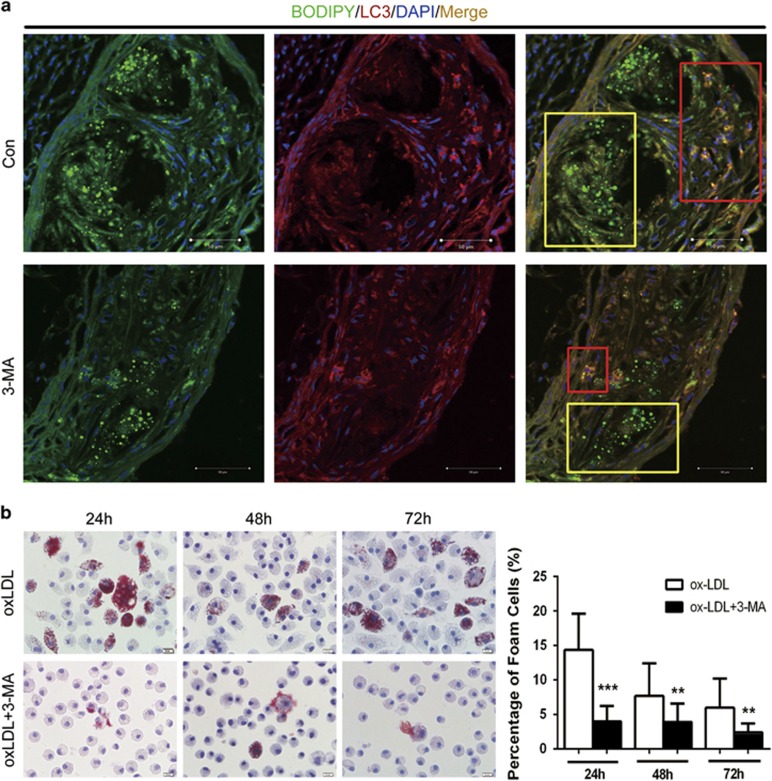
3-MA decreased foam cell macrophage formation *in vitro* and accumulation of lipid droplets within atherosclerotic plaque. (**a**) Representative images of BODIPY (green) and LC3 (red) in aortic plaque from control mice and 3-MA-treated mice. Red boxes highlight LC3-positive area and yellow boxes LC3-negative areas. Scale bar, 50 *μ*m. (**b**) Cells were preincubated with 3-MA for 30 min, then stimulated with 25 *μ*g/ml oxLDL for foam cell induction. Oil Red O staining was performed at different times after oxLDL stimulation. The proportion of Oil Red O-positive cells from three independent experiments was calculated and data are mean±S.D. **P<0.01, ***P<0.001, unpaired two-tailed Student's *t*-test

Recent research indicates that autophagy can mediate the breakdown of lipids in LDs, reduce macrophage foam cells and further inhibit atherogenesis.^15, 31^ To determine whether long-term treatment with 3-MA benefits atherosclerosis protection via autophagy, we co-stained plaque areas for LDs and LC3. The number of LDs within plaque was markedly lower with 3-MA than control treatment (Figure 4a). Furthermore, the number of LDs was reduced in LC3-positive regions but was higher in LC3-negative regions, which supports that autophagy promotes the breakdown of LDs in plaque. Furthermore, 3-MA greatly reduced macrophage formation at 24, 48 and 72 h after oxLDL stimulation *in vitro* (Figure 4b). 3-MA exposure retained mouse peritoneal macrophages in a round shape with less lipid burden under oxLDL challenge as compared with the spindle shape and foamy morphology of characteristic foam cells with oxLDL treatment alone (Figure 4b). The lipid burden of macrophages without 3-MA treatment was gradually decreased at 72 h as compared with 24 h. Oil Red O-positive-stained LDs moved radially from the center to the edge of cell membranes over time. We thought cells were undergoing recovery after oxLDL challenge, but we did not see this phenomenon in 3-MA-treated macrophages.

### 3-MA inhibited the viability of oxLDL-stimulated macrophages *in vitro*

**Figure 5:**
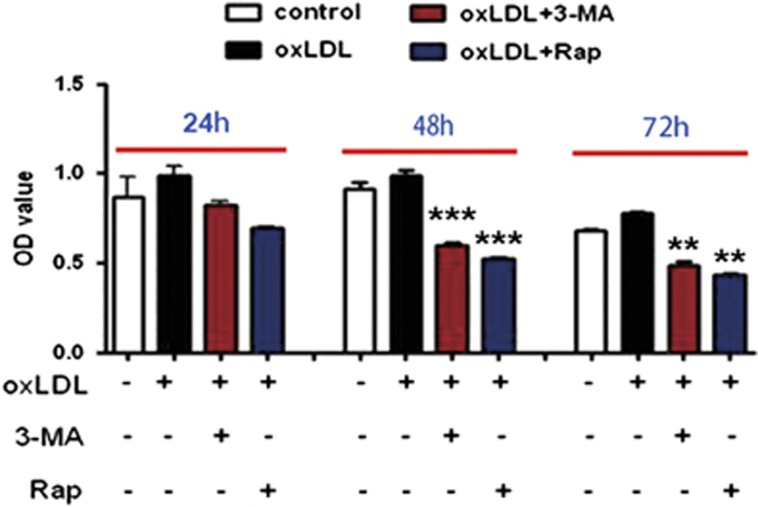
3-MA inhibited viability of oxLDL-stimulated macrophages *in vitro*. Cells were preincubated with 3-MA or rapamycin for 30 min, and then stimulated with 25 *μ*g/ml oxLDL. CCK-8 assay was performed at different times after oxLDL stimulation to measure the viability of microphages. Data from three independent experiments are mean±S.D. (*n*=3 replicates). **P<0.01, ***P<0.001, unpaired two-tailed Student's *t*-test

Previous research reported that the mTOR inhibitor, everolimus, can selectively deplete macrophages in atherosclerotic plaques by inducing autophagy.^21, 22^ 3-MA-treated macrophages did not show the classical morphology of foam cells and recovery tendency after oxLDL stimulation *in vitro* (Figure 4b), so we wondered whether 3-MA could eliminate autophagy-induced macrophages by promoting autophagy, similar to everolimus. In Figure 2b, 3-MA treatment significantly reduced macrophage content in plaque lesions. Furthermore, 3-MA, as well as rapamycin, the classical mTOR inhibitor, time-dependently inhibited cell viability as compared with controls (Figure 5).

### 3-MA improved the anti-inflammatory microenvironment in atherosclerotic plaque

**Figure 6:**
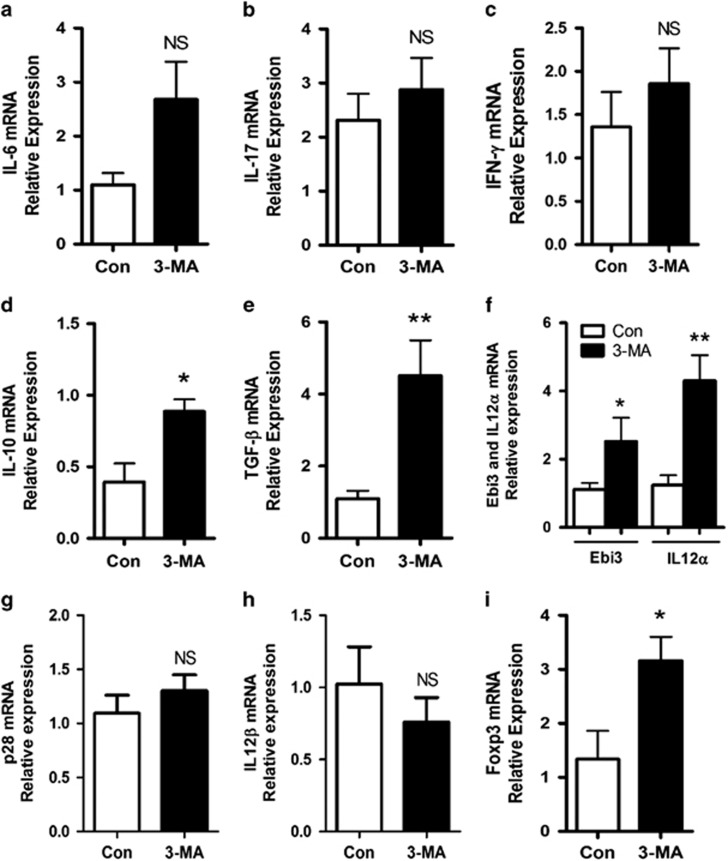
3-MA improved anti-inflammatory microenvironment in atherosclerotic plaque. Quantitative RT-PCR analysis of messenger RNA (mRNA) expression of IL-6, IL-17, interferon-gamma (IFN-*γ*), IL-10, TGF-*β*, Ebi3, IL-12*α*, p28, IL-12*β* and Foxp3 in the aorta of control mice (4 mice from 14 control mice) and 3-MA-treated mice (6 mice from 16 3-MA-treated mice). Data from three replicates in three independent experiments are mean±S.D. *P<0.05, **P<0.01, unpaired two-tailed Student's *t*-test

Previous research revealed that autophagy is associated with regulation of the inflammatory microenvironment in plaque.^14, 23^ Therefore, we detected the impact of 3-MA on the gene expression of cytokines in arterial walls containing plaques. In contrast to our prediction, 3-MA treatment had no effect on the expression of proinflammatory cytokines such as IL-6, IL-17 A and interferon-gamma (Figures 6a–c) as compared with controls, but did increase the expression of anti-inflammatory cytokines such as IL-10 and transforming growth factor-beta (TGF-*β*; Figures 5d and e), as well as IL-35 (Figure 5f), consisting of the Ebi3 subunit and IL-12*α* subunit. As Ebi3 combined with p28 can form IL-27 and IL-12*α* with IL-12*β* from IL-12, we also tested p28 and IL-12*β* expression (Figures 5g and h), and found no change in expression of these two subunits between 3-MA-treated mice and controls, so the increased level of Ebi3 and IL-12*α* level should indicate upregulation of IL-35 but not IL-27 or IL-12. As IL-10, TGF-*β* and IL-35 are effector molecules of regulatory T cells (Tregs), we further analyzed the expression of Foxp3, a specific transcription factor for Tregs, and found that 3-MA treatment significantly upregulated Foxp3 expression (Figure 5i).

## Discussion

Recent studies demonstrate that autophagy is involved in the pathogenesis of atherosclerosis and has become a potential therapeutic target for treating atherosclerosis. In this study, we tested the effect of two commonly used autophagy inhibitors, 3-MA and LY294002, in atherosclerosis. 3-MA treatment markedly reduced the size of plaque in the aortic root and *en face-*stained lesions in the aortic arch, thoracic aorta and abdominal aorta. 3-MA also significantly increased the content of SMCs, but decreased that of lipids and number of macrophages in plaque. In addition, the number of apoptotic cells was reduced by 3-MA. Thus, 3-MA inhibits the formation of atherosclerotic lesions and increases plaque stabilization. In addition, the low concentration of 3-MA in aqueous solution we used was previously found to have no side effects,^28^ and even slightly improved liver function, as shown by low level of AST.

Compared with 3-MA, LY294002 did not confer significant protection in atherosclerosis development perhaps because of the dose of LY294002 we used for *in vivo* treatment or the molecular weight of LY294002 (307.35) being higher than that of 3-MA (149.15), which may result in a different distribution pattern and function efficiency for LY294002 and 3-MA.

We further explored the underlying mechanism of the athroprotective effect of 3-MA. 3-MA has been widely used as an autophagy inhibitor for many years, but recent reports indicate that it has dual role in regulating autophagy. 3-MA suppresses the early stage of autophagy under nutrient deprivation by its inhibitory effect on class III PI3K activity, but promotes autophagy flux under nutrient-rich conditions with prolonged treatment by its inhibitory effect on class I PI3K activity.^24, 25^ Thus, 3-MA-modulating autophagy is complicated and variable, and depends on different conditions.

To determine whether autophagy has a role in 3-MA-mediated atherosclerosis protection, we analyzed autophagy level both *in vivo* and *in vitro*. *In vitro*, 3-MA treatment significantly promoted macrophage autophagy under oxLDL challenge and reduced oxLDL-induced foam cell formation, which is consistent with our *in vivo* finding that 3-MA decreased the lipid content and number of LDs in atherosclerotic plaque. These results agree with the previous findings of increased autophagy facilitating the breakdown of LDs and reducing foam cell formation, thereby suppressing atherosclerosis.^15, 32^ We found that 3-MA attenuated macrophage viability *in vitro*, and 3-MA-treated macrophages barely changed into a foamy morphology with oxLDL stimulation, whereas most 3-MA non-treated cells were spindle shape and showed lipid efflux to some extent for cell recovery. Earlier studies showed that high level of autophagy triggered by an autophagy inducer or certain stress conditions could result in autophagic cell death, a form of cell death that can be independent of apoptosis and the caspase pathway.^13, 21, 22, 33^ In the current study, 3-MA likely promoted macrophage autophagy under atherosclerosis challenge, which facilitated the suppression of foam cell formation and reduced apoptosis *in vivo*. However, prolonged active autophagy can induce blockage of protein synthesis and degradation of cytoplasmic components,^22^ which results in autophagic cell death of macrophages. Thus, we found lower protein level of LC3 within the vascular wall in mice after 8 weeks of 3-MA treatment. The decreased tendency of pS6 level with 3-MA treatment may be evidence that the class I PI3K pathway was inhibited by 3-MA and led to augmented autophagy. Collectively, our data show that 3-MA facilitates the breakdown of LDs, reduces foam cell formation and depletes foam cell macrophages by regulating autophagy activity. Further investigation is needed to unmask the diverse regulations of 3-MA on autophagy of atherosclerosis-associated cells *in vivo* and *in vitro*.

In addition, the anti-atherosclerotic effect of 3-MA may be attributed to the modulation of inflammatory responses. Previous studies have clarified the atheroprotective role of anti-inflammatory cytokines, including IL-10, TGF-*β* and IL-35.^34, 35, 36, 37^ Here we found upregulation of these suppressive molecules in the artery wall of 3-MA-treated mice, which suggests that the treatment benefited an anti-inflammatory microenvironment within the lesion area. Furthermore, the upregulation of Foxp3 suggests that Tregs, the dominant regulator of negative immune responses and critical origin of suppressive molecules, were potentially enhanced by 3-MA in the vessel wall. Accumulating evidence demonstrates that Tregs have a protective role in atherosclerosis and may be a new promising target in atherosclerosis.^38, 39, 40, 41^ Moreover, several studies have found that inhibition of the PI3K pathway is essential for the development of Foxp3+ natural Tregs and conversion of naive CD4+ T cells to induced Tregs. Further study is required to determine whether the elevated Foxp3 level we found results from 3-MA-blocked PI3K.

Taken together, we provide strong evidence that 3-MA can inhibit atherosclerosis development and improve plaque stabilization in HFD-fed ApoE^−/−^ mice. Pleiotropic effects, including modulating macrophage autophagy, suppressing foam cell formation and promoting an anti-inflammatory immune microenvironment, reveal the atheroprotective role of 3-MA. Although the underlying mechanism is still unclear, we shed new light on developing novel anti-atherosclerotic therapies by targeting 3-MA and its derivatives.

## Materials and Methods

### Animals

Male ApoE^−/−^ mice (8 weeks old, 17–22 g) were purchased from Beijing University and were housed at a constant temperature (24 °C) room, under a 12 h dark/12 h light cycle in a pathogen-free environment in the Animal Care Facility of Shandong University Medical School according to institutional guidelines. All mice had access to water and regular mouse food *ad libitum*. All efforts were made to minimize animal suffering as well as the number of animals used. All animal studies were approved by the Animal Care and Utilization Committee of Shandong University, China.

### *In vivo* application of 3-MA and LY294002

ApoE^−/−^ mice were fed a HFD starting from 8 to 17 weeks old. We performed the first experiment with three groups of randomly divided mice: (1) control (PBS, i.p., *n*=6); (2) 3-MA (30 mg/kg, Sigma-Aldrich, St. Louis, MO, USA, *n*=6, according to previous research;^42^ and (3) LY294002 (0.3 mg/kg, Sigma-Aldrich, *n*=6, according to previous research^43^). Each animal received i.p. injection twice a week for 8 weeks. Both 3-MA and LY294002 were dissolved in PBS and kept at −20 °C. We heated the 3-MA solution to 60 °C immediately each time before injection. One week after the last treatment, mice were killed for further study.

From preliminary data, we confirmed the atheroprotective effect of 3-MA *in vivo* and further studied the potential mechanism involved in it. Another intervention study was carried out with two groups of mice: (1) control (PBS, i.p., *n*=8); and (2) 3-MA (30 mg/kg, Sigma-Aldrich, *n*=10). All treatments and time schedule were as for the first study.

### Measurements of clinical biochemical parameters

Blood samples were collected by retro-orbital bleed. After 30-min incubation at room temperature, samples were centrifuged (4 °C, 2500 r.p.m., 20 min) to obtain serum and stored at −80 °C until analysis. Chemical parameters were detected by an automated chemically modified technique (Roche Modular DPP System, Roche, Basel, Switzerland): (1) the lipid panel including TC, total TGs, LDL cholesterol and HDL cholesterol; (2) liver function including alanine aminotransferase and AST; and (3) kidney function including serum creatinine and blood urea nitrogen.

### *En face* staining analysis

Six mice from each group were killed, and aortas from the heart to the iliac arteries were dissected, soaked overnight in 4% paraformaldehyde for fixation, excised longitudinally and stained with 0.5% Oil Red O (O0625, Sigma-Aldrich) for 2 h, and then pinned flat on a black surface by using Minutien (Fine Science Tools, Inc, Foster City, CA, USA). Aortas were imaged by an EPSON Perfection 2450 photo scanner (EPSON, Long Beach, CA, USA) and analyzed using Image Pro-Plus 6.0. (Media Cybernetics, Rockville, MD, USA)

### Histopathology, immunohistochemistry and immunofluorescence

After mice were killed, aortic root vessels were fixed in 4% paraformaldehyde overnight and then embedded in optimal cutting temperature compound (Sakura Finetek, Torrance, CA, USA). Serial cryosections (7 *μ*m thickness) were cut along the aortic root specimens using a cryotome (HM550, Thermo Scientific, Rockford, IL, USA). At least three transverse sections (spaced ~50 *μ*m) from each aortic root were stained with H&E for histopathology analysis. Lipid content in plaque area was evaluated by Oil Red O staining and collagen content by Sirius red staining. To detect the proportion of macrophages and SMCs, corresponding cryosections on separate slides underwent immunohistochemistry (IHC) staining with a rat anti-mouse macrophage-specific antibody (MOMA-2; MCA519G, AbD Serotec, Oxford, UK) or a rabbit anti-mouse polyclonal antibody for smooth muscle actin (ab5694, Abcam, Hong Kong, China). pS6 was detected by rabbit anti-mouse Phospho-S6 Ribosomal Protein (Ser235/236) monoclonal antibody (#4858, Cell Signaling Technology, Beverly, MA, USA). Negative controls for the specificity of each immunohistochemical reaction involved replacing the primary antibody with isotype IgG (A7016, Beyotime, Shanghai, China). Apoptotic cells in aortic root sections were detected using an *in situ* cell death detection kit (Roche, Nutley, NJ, USA). All lesions were observed under an Olympus microscope (IX71, Olympus Corp., Osaka, Japan). For detecting autophagosome and lipid storage in plaque, cryosections were stained for LC3 by a rabbit anti-LC3 antibody (1:100 dilution; 2775 S, Cell Signaling Technology), and LDs were stained with BODIPY 493/503 (D-3922, Thermo Fisher Scientific, Lafayette, CO, USA). After an overnight incubation of primary antibodies at 4 °C, sections were incubated with Alexa 555-conjugated secondary antibody (A-21429, Life Technologies, Gaithersburg, MD, USA) for LC3 and Alexa 488-conjugated secondary antibody (1:1000 dilution; A-11034, Life Technologies) for BODIPY. After 1 h, slides were washed with PBS. Nuclei were stained with 4′,6-diamidino-2-phenylindole (300 nM; D1036, Life Technologies) for 5 min. Images were detected by confocal laser scanning microscopy (LSM780, Carl Zeiss, Jena, Germany). All plaque areas with positive staining were analyzed using Image Pro-Plus 6.0 (Media Cybernetics, Rockville, MD, USA).

### Real-time PCR

Total RNA in the arterial wall (four mice each from the 3-MA or control group) was isolated by the Trizol reagent method (15596-026, Life Technologies). Next, 1 *μ*g of total RNA was used for reverse transcription by the use of RT-PCR quick master mix kit (PCR-311, Toyobo, Osaka, Japan). Real-time PCR was performed with equal amounts of cDNA in triplicate and Ultra SYBR Mixture (CW0956, CW Bio, Beijing, China), detected using the CFX 96 Real-Time Detection System (Bio-Rad, Richmond, CA, USA). The sequences of sense and antisense primers with related genes are in Supplementary Table S1. Data for relative molecule expression were presented using the 2^ΔΔCt^ method. Data are reported as fold change in the experimental group normalized to an endogenous reference gene (18 S) and relative to the control group.

### Mouse primary peritoneal macrophage culture and foam cell formation assay

Mouse primary peritoneal macrophages were obtained from C57BL/6 mice 3 days after i.p. injection of 3% thioglycollate broth medium. Isolated macrophages were plated at 5 × 10^5^ cells per ml in Dulbecco's modified Eagle medium (DMEM) containing 10% fetal bovine serum (FBS) and cultured at 37 °C with 5% CO_2_.For foam cell formation assay, mouse primary peritoneal macrophages were pretreated of 5 mM 3-MA (3-MA group) or PBS (control group) in DMEM containing 10% FBS for 30 min at 37 °C, and then 50 *μ*g/ml oxLDL was added to medium of both 3-MA and control groups to induce foam cell formation. Cells without any treatment were used as a negative control. Cells were extensively washed for Oil Red O staining or total protein extraction 24, 48 or 72 h after treatment.

### Cell viability detected by CCK-8 assay

Cell viability was tested using the cell counting kit-8 (CCK-8) assay. Mouse primary peritoneal macrophages were seeded in 96-well cell culture plates at 5 × 10^3^ cells per well and divided into four groups for treatment: no treatment, oxLDL treatment only, 30 min 3-MA preincubation (5 mM) before oxLDL treatment and 30 min rapamycin preincubation (100 nM) before oxLDL treatment. Cells were cultured at 37 °C for 24, 48 and 72 h. At each time, cells were washed. An amount of 10 *μ*l CCk-8 solution was added to each well for incubation at 37 °C for 1 h. The absorbance of each well was measured at 450 nm. Each treatment was performed in triplicate.

### Western blot analysis

Cultured cells and fragments of the arterial wall were extracted by RIPA Lysis Buffer (Beyotime) containing protease and phosphatase inhibitors. Quantified samples with 30 *μ*g total protein were separated on 12% SDS-ployacrylamide gel electrophoresis and transferred onto polyvinylidene difluoride membranes (Bio-Rad, Wattford, UK), which were blocked with 2% (wt/vol) bovine serum albumin in Tris-buffered saline solution containing 0.1% Tween-20 for 1 h. Membranes were incubated overnight at 4 °C with the primary antibodies for LC3 (1:1000 dilution; 2775 S) and SQSTM1/p62 monoclonal antibody (1:1000 dilution; 8025 S, both from Cell Signaling Technology), mouse monoclonal antibody against ACTIN (1:2000 dilution; sc-7210, Santa Cruz Biotechnology, Santa Cruz, CA, USA) and GAPDH, followed by peroxidase-conjugated secondary antibody (A0208, Beyotime) for 1 h at room temperature. After washing, signals were visualized using Super Signal West Pico Chemiluminescent Substrate (Pierce Biotechnology, Rockford, IL, USA). Western blots were prepared at least three times for each sample.

### Statistical analysis

Statistical analysis involved use of GraphPad Prism 6.0 (GraphPad Software, San Diego, CA, USA). Data are expressed as mean±S.E.M. Unpaired Student's *t*-test was used to compare data in different groups. Two-way ANOVA was used to analyze grouped data over time. *P*<0.05 was considered statistically significant.
