# Supplementary figures and images for: Systemic application of 3-methyladenine markedly inhibited atherosclerotic lesion in ApoE−/− mice by modulating autophagy, foam cell formation and immune-negative molecules
Source: Cell Death Dis. 2016 Dec 1;7(12):e2498–. doi: 10.1038/cddis.2016.376 (PMC5260998; doi:10.1038/cddis.2016.376)

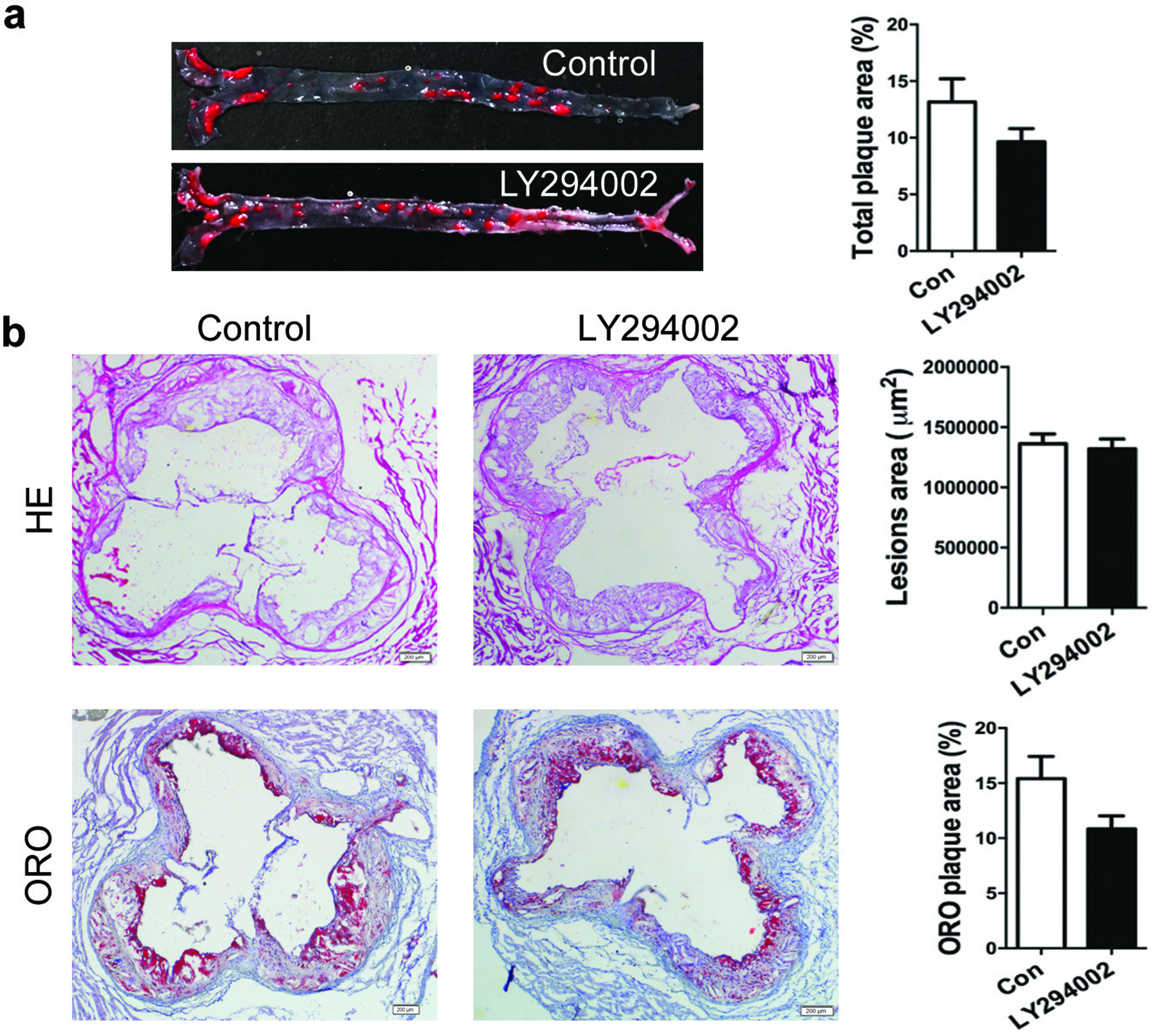

Supplement: Supplementary Figure 1 [file cddis2016376x1.tif]

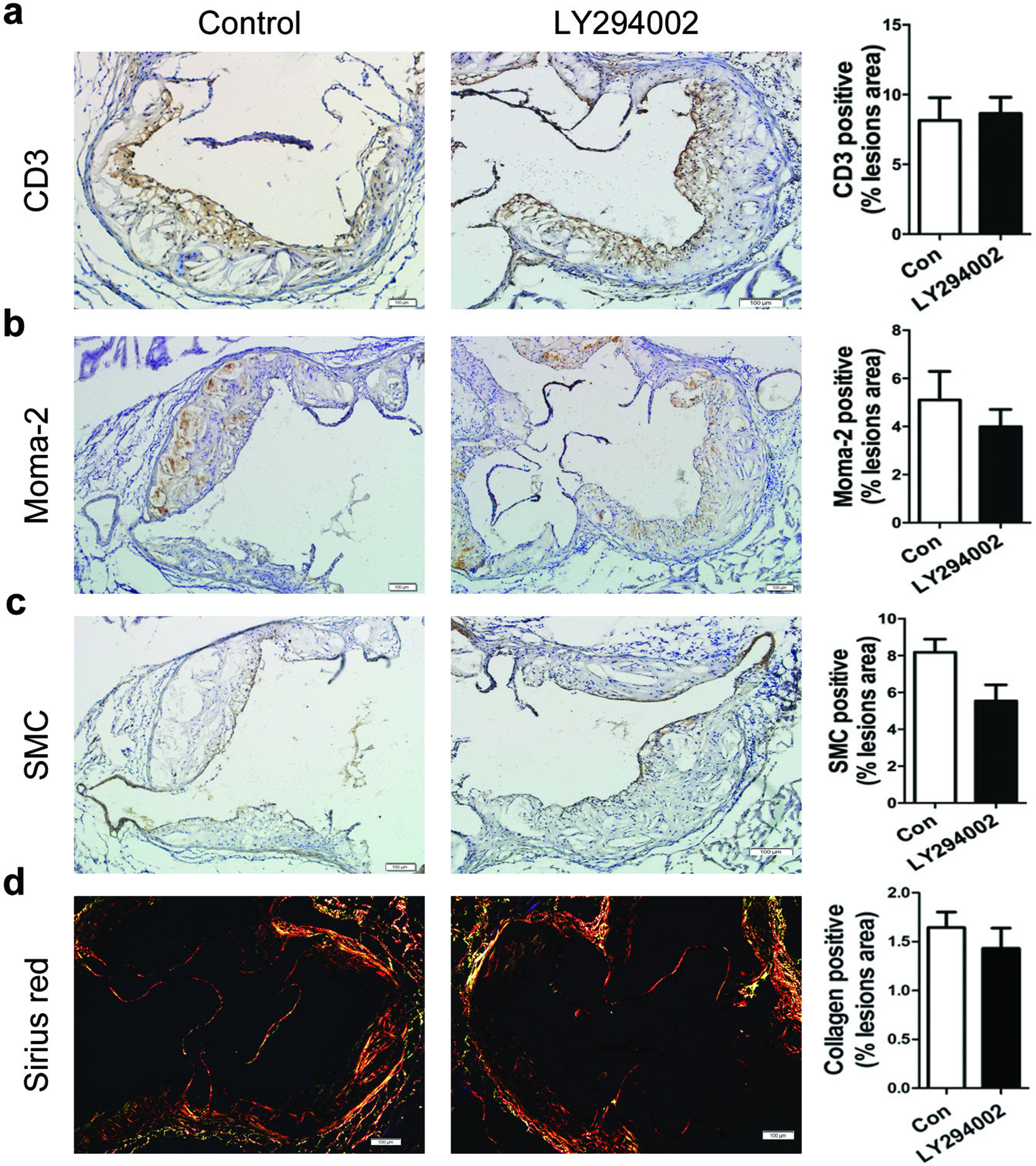

Supplement: Supplementary Figure 2 [file cddis2016376x2.tif]

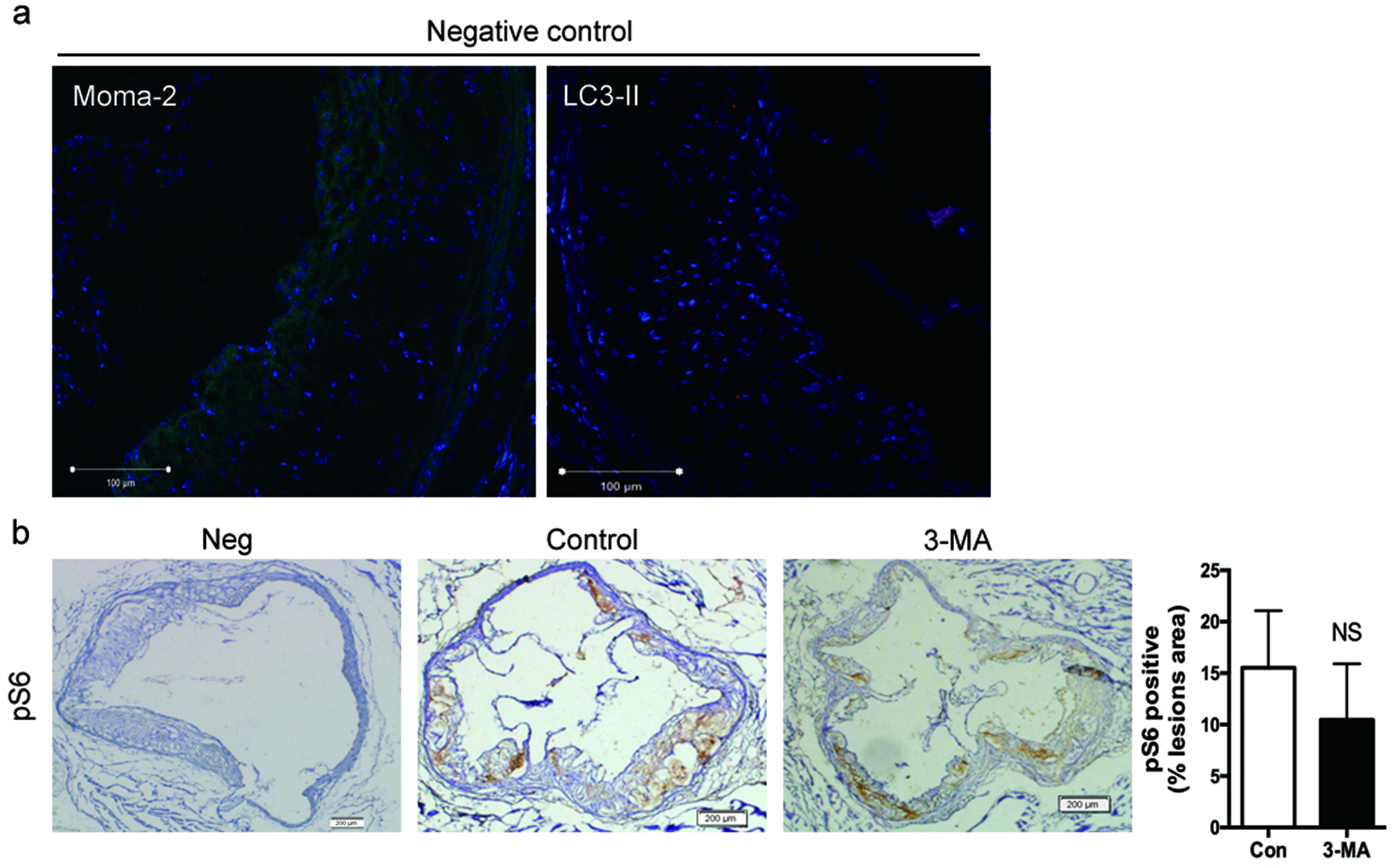

Supplement: Supplementary Figure 3 [file cddis2016376x3.tif]
