# Supplementary material for: Systemic application of 3-methyladenine markedly inhibited atherosclerotic lesion in ApoE−/− mice by modulating autophagy, foam cell formation and immune-negative molecules
Source: Cell Death Dis. 2016 Dec 1;7(12):e2498–. doi: 10.1038/cddis.2016.376 (PMC5260998; doi:10.1038/cddis.2016.376)
Supplement: Supplementary Tables [file cddis2016376x4.pdf]

Supplemental table

Table 1. PCR Primers

Gene	Forward 5'->3'	Reverse 5'->3'
IL-10	GCCAGAGCCACATGCTCCTA	GATAAGGCTTGGCAACCCAAGTAA
TGF-β	GTGTGGAGCAACATGTGGAACTCTA	CGCTGAATCGAAAGCCCTGTA
EBi3	AGCAGCCTCCTAGCCTTTGTGG	GAGTTCCTGAGGGTGAAAGTCGTG
IL12α	AGCGTTCCAACAGCCTCAC	CTCTGGCCGTCTTCACCAT
p28	GCACAGGCACCTCCGCTTTCA	GCAGCAGCAGGTCCCGAACAG
IL12β	TGTCACCAGCAGTTGGTCATCTC	CTCACTGCTCTGGTCCAAGGTC
Foxp3	CCCAGGAAAGACAGCAACCTT	TTCTCACAACCAGGCCACTTG
IL-17A	CTGATCAGGACGCGCAAAC	TCGCTGCTGCCTTCACTGTA
IL-6	CCACTTCACAAGTCGGAGGCTTA	CCAGTTTGGTAGCATCCATCATTTC
IFN-γ	ATGTATTGCTTTGCGTTGGACA	TCAATAGCAACAAAAAGAAACGAGAT
18S	AACCCGTTGAACCCCATT	CCATCCAATCGGTAGTAGCG

Table 2. Body weight and plasma lipid levels with or without 3-MA treatment

Mice	TGs	TCH	HDL	LDL	Body Weight
Control (n=14)	1.91±0.36	34.49±2.47	9.23±0.97	31.28±1.61	26.63±0.34
3-MA (n=16)	1.99±0.28	35.21±3.41	9.49±1.45	30.33±1.89	29.39±0.74
LY294002 (n=6)	1.63±0.17	30.48±2.08	10.00±0.52	26.43±1.85	29.20±0.52

Data are expressed as mean ±SEM.

Table 3 Liver function and kidney function of control mice and 3-MA treated mice

Mice	ALT	AST	BUN	Cr
Control (n=14)	83.20±33.49	385.8±25.99	7.52±0.71	4.60±1.29
3-MA (n=16)	45.33±5.47	278.7±22.51*	8.35±0.33	4.50±0.76
LY294002 (n=6)	65.33±11.69	345.7±26.28	8.18±0.46	3.17±0.83

Data are expressed as mean ±SEM. *p<0.05
